# Impaired Bile Acid Homeostasis in Children with Severe Acute Malnutrition

**DOI:** 10.1371/journal.pone.0155143

**Published:** 2016-05-10

**Authors:** Ling Zhang, Wieger Voskuijl, Marialena Mouzaki, Albert K. Groen, Jennifer Alexander, Celine Bourdon, Alice Wang, Christian J. Versloot, Valeria Di Giovanni, Ronald J. A. Wanders, Robert Bandsma

**Affiliations:** 1 Physiology and Experimental Medicine Program, Research Institute, The Hospital for Sick Children, Toronto, Canada; 2 Department of Paediatrics & Children Health, College of Medicine, P/Bag 360, University of Malawi, Blantyre, Malawi; 3 Global Child Health Group, Emma Children’s Hospital/AMC, Amsterdam, The Netherlands; 4 Department of Pediatrics, Division of Gastroenterology, Hepatology and Nutrition, The Hospital for Sick Children, Toronto, Canada; 5 Department of Pediatrics, Center for Liver, Digestive and Metabolic Diseases, University of Groningen, University Medical Center Groningen, Groningen, The Netherlands; 6 Laboratory Genetic Metabolic Diseases, Departments of Pediatrics and Clinical Chemistry, Academic Medical Center, Amsterdam, The Netherlands; 7 Centre for Global Child Health, The Hospital for Sick Children, Toronto, Canada; Texas A&M University, UNITED STATES

## Abstract

**Objective:**

Severe acute malnutrition (SAM) is a major cause of mortality in children under 5 years and is associated with hepatic steatosis. Bile acids are synthesized in the liver and participate in dietary fat digestion, regulation of energy expenditure, and immune responses. The aim of this work was to investigate whether SAM is associated with clinically relevant changes in bile acid homeostasis.

**Design:**

An initial discovery cohort with 5 healthy controls and 22 SAM-patients was used to identify altered bile acid homeostasis. A follow up cohort of 40 SAM-patients were then studied on admission and 3 days after clinical stabilization to assess recovery in bile acid metabolism. Recruited children were 6–60 months old and admitted for SAM in Malawi. Clinical characteristics, feces and blood were collected on admission and prior to discharge. Bile acids, 7α-hydroxy-4-cholesten-3-one (C4) and FGF-19 were quantified.

**Results:**

On admission, total serum bile acids were higher in children with SAM than in healthy controls and glycine-conjugates accounted for most of this accumulation with median and interquartile range (IQR) of 24.6 μmol/L [8.6–47.7] compared to 1.9 μmol/L [1.7–3.3] (*p* = 0.01) in controls. Total serum bile acid concentrations did not decrease prior to discharge. On admission, fecal conjugated bile acids were lower and secondary bile acids higher at admission compared to pre- discharge, suggesting increased bacterial conversion. FGF19 (Fibroblast growth factor 19), a marker of intestinal bile acid signaling, was higher on admission and was associated with decreased C4 concentrations as a marker of bile acid synthesis. Upon recovery, fecal calprotectin, a marker of intestinal inflammation, was lower.

**Conclusion:**

SAM is associated with increased serum bile acid levels despite reduced synthesis rates. In SAM, there tends to be increased deconjugation of bile acids and conversion from primary to secondary bile acids, which may contribute to the development of liver disease.

## Introduction

### Background on severe acute malnutrition

Severe acute malnutrition (SAM) is a major cause of death in children under the age of five years [1]. The pathophysiology of SAM is poorly understood but has been associated with liver disease, most commonly hepatic steatosis. In addition, SAM is associated with significant intestinal disturbances, such as mucosal atrophy [2] and impaired intestinal epithelial barrier function [3, 4]. Also, lipid [3, 5] and carbohydrate [6] maldigestion and malabsorption are common in SAM. Two studies recently described profound SAM-associated changes in the intestinal microbiota [7, 8]. Considering that children hospitalized with complicated SAM have a mortality rate of up to 30%, it is crucial to better understand the pathophysiological underpinnings of SAM [9–11].

The impaired hepatic and intestinal functions together with the altered microbiota seen in SAM patients could be related to changes in bile acid homeostasis. Bile acids are synthesized in hepatocytes and the rate-limiting step for their synthesis is controlled by *Farnesoid X Receptor* (FXR). FXR is a nuclear receptor mostly expressed in the liver and intestines that is activated by BAs which can be modulated by intestinal microbiota [12]. In the intestine, FXR induces *Fibroblast growth factor-19* (FGF19) transcription when BA levels in the ileum are elevated [13]. FGF19 then enters circulation and negatively regulates bile acid synthesis in the liver by inhibiting the rate-limiting enzyme *cholesterol 7α-hydroxylase* (CYP7A1) through *Fibroblast growth factor receptor-4* mediated signaling [14]. The intermediate *7α-Hydroxy-4-cholesten-3-one* (C4) is produced while synthesizing bile acids from 7α-hydroxycholesterol, which is itself produced from cholesterol by hepatic CYP7A1 [15]. C4 is a measure of bile acid synthesis rate.

Following BA synthesis, primary bile acids chenodeoxycholic acid (CDCA) and cholic acid (CA) are conjugated with glycine or taurine and released in the small intestine to facilitate lipid emulsification, digestion and absorption. There, they are deconjugated and modified into secondary bile acids by intestinal microbiota leading to deoxycholic acid (DCA, from CA) and lithocholic acid (LCA, from CDCA) synthesis [16]. Bile acids are intrinsically toxic to cells mainly due to their inherent detergent and membrane disruptive properties, which can contribute to hepatotoxicity, impaired epithelial barrier function and increased bacterial translocation [17, 18]. Bile acids have both hydrophobic and hydrophilic properties with hydrophobic strength ordered as LCA>DCA>CDCA>CA [19]. Hydrophobic bile acids (such as LCA) can contribute to intestinal inflammation [19]. Considering that SAM is associated with dysbiosis and that intestinal microbiota regulate bile acid synthesis, deconjugation and metabolism, it is important to understand the role of bile acids in the development of SAM-related complications.

There is paucity of data on the topic of bile acid homeostasis in SAM [20, 21]. Understanding their role would provide important insight into the pathogenesis of malnutrition and its complications but more importantly may suggest future therapeutic targets. We therefore aimed to address the following questions: 1. Is SAM associated with changes in the homeostasis of bile acids? 2. If so, does nutritional rehabilitation restore bile acid homeostasis in patients with SAM? 3. Do changes in bile acid metabolism relate to markers of intestinal or hepatic inflammation?

## Subjects and Methods

### Patient recruitment and study design

Recruited children were 6 months to 5 years of age and hospitalized for SAM at Queen Elisabeth Central Hospital in Blantyre, Malawi. The discovery cohort was composed of 22 SAM-patients and 5 healthy controls recruited for a study evaluating glucose homeostasis. The follow up cohort was composed of 40 SAM-patients recruited for a prospective study aimed at investigating the outcome of three WHO recommended standard rehabilitation diets (ISRCTN 13916953). Exclusion criteria were suspected tuberculosis, confirmed malaria or lack of sufficient serum for analyses. Patients with kwashiorkor, marasmus or HIV were included. Healthy controls were recruited at hospital discharge having recovered from minor orthopedic or suspected viral respiratory problems. Written consent was obtained from care givers using paper consent forms in accordance with the protocol approved by the College of Medicine Research Ethics Committee, Malawi, and the Research Ethics Board (REB number 1000048167) at the Hospital for Sick Children, Toronto, Canada.

### Inpatient treatment

As per protocol, all hospitalized patients received broad-spectrum antibiotics on admission and in addition, children with HIV continued with their outpatient treatment of oral cotrimoxazole prophylaxis for *pneumocystis jiroveci* pneumonia. Nutritional management of patients was initially based on the standard WHO protocol, which recommends a low-energy (75–100 kcal energy/kg/day), low-protein (F75; 1.2g protein/kg/day) liquid diet until improvement of appetite and, for children with kwashiorkor, loss of edema [22]. Subsequently, calorie intake was increased and children were either given Ready-to-use therapeutic food (RUTF) supplemented with F75 if required, or a more calorie dense formula, *i*.*e*., F100.

### Clinical data and specimen collection

Baseline clinical data were recorded for weight, height, MUAC and presence and degree of edema and weight was then monitored daily. Unfasted blood samples were collected within 24 hours of admission for the discovery cohort. We obtained additional samples in the follow up cohort where both unfasted blood and stool were collected upon admission and 3 days after clinical stabilization just prior to discharge. Serum aliquots and fecal samples were frozen and stored at -80°C until analysis.

### Serum and fecal bile acid analysis

Serum bile acid concentrations of the discovery cohort were determined by high-performance liquid chromatography (HPLC) negative ion electrospray tandem mass spectrometry as described previously [23]. Those of the follow up cohort were measured by liquid chromatography–mass spectrometry (LC-MS) using the Biocrates^®^ Life Sciences Bile Acids Kit (Biocrates, Innsbruck, Austria) as per manufacturer’s instructions using an Agilent UHPLC 1290 LC system coupled to an ABSciex QTRAP 5500 in negative ESI MRM mode. The same kit was used to analyze fecal bile acids. Samples were lyophilized, reconstituted with 75% ethanol and homogenized then vortexed for 5 minutes and centrifuged at 20,000 g for 10 minutes. The resulting supernatant was used for bile acids measurement.

### Determination of hepatic bile acid synthesis 7α-hydroxy-4-cholesten-3-one (C4)

Serum C4 measurement was performed by LC-MS-MS. C4 was extracted using a salting-out method as previously described [24, 25]. Serum samples were prepared alongside of authentic C4 standards. Briefly, 100 μL of serum was diluted with 200 μL of distilled water, to which 5 ng of d7-C4 (used as internal standard) and 500 μL of acetonitrile were added. Then, 100 mg of ammonium sulfate was added, tubes vortexed for 1 minute and centrifuged at 2,000 g at 4°C for 5 minutes. The supernatant acetonitrile phase was collected and dried under nitrogen at 35°C. The residues were reconstituted with 200 μL methanol, vortexed for 1 minute, incubated for 10 minutes and centrifuged at 20,000 g for 5 minutes. Clear supernatants were transferred to 250 μL inserts for LC-MS-MS analysis. The LC-MS-MS system consisted of an Agilent UHPLC 1290 LC system coupled to an ABSciex QTRAP 5500 in positive ESI MRM mode. Chromatographic separation was performed using a XB-C18 kinetex column (50 x 3.0 mm, 2.6 μm; Phenomenex) operated at a flow rate of 800 μL/minute and eluted isocratically with a mobile phase consisting of acetonitrile/water (98/2, v/v) with 0.1% trifluoroacetic acid. Quantitation of C4 was achieved by comparing the deuterium-to-protium ratio of the samples with a standard using the Analyst 1.6 software.

### Determination of fibroblast growth factor (FGF) 19 and calprotectin

Serum FGF-19 levels were determined using sandwich enzyme-linked immunosorbent assay (R&D Systems, Minneapolis, MN, USA) as per manufacturer’s instructions. Samples (100 μl) were diluted (1:2) using the Calibrator Diluent RD5P (1X) supplied with the kit. Fecal calprotectin was measured by standard enzyme-linked immunoabsorbent assay (Bühlmann Laboratories, Schönenbuch, Switzerland). Alanine aminotransferase (ALT) concentrations were determined using standard laboratory analyses.

### Statistical analysis

Data were expressed as median (IQR). Mann-Whitney test was used to compare controls and SAM-patients of the discovery cohort whereas Wilcoxon signed rank test for paired samples was used to compare admission and prior to discharge in the follow up cohort. Differences in calprotectin at admission and pre-discharge were tested for significance with mixed effects models with patients set as random factor to account for missing data and within patient repeated measures. Spearman’s correlations were used to determine relationships between non-parametric data. The significance threshold was set at *p*-value<0.05 for all statistical tests. Analyses were conducted with SPSS 22.0 software (IBM Corporation) and R statistical software (Version 3.2.2). Plots were generated using ggplot2 [26].

## Results

The clinical characteristics of all study participants (n = 75) are detailed in Table 1. Children were on average 24.8 ± 11.7 months old. A substantial proportion of the malnourished children (44%) were HIV positive and more than 60% presented with edema. Electrolyte imbalance was apparent with approximately 20% of patients showing either hypo- or hypernatremia. Levels of ALT, a marker of liver injury, were increased in SAM-patients with a median (IQR) of 46 [27–64] IU/I compared to14 [9–17] IU/I in healthy controls (*p*<0.001).

**Table 1:** Characteristics of all patients at admission.

	Control	Malnourished
	(n = 5)	(n = 70)
Age (month)	26 ±10.3	23.7 ±1.4
Female, n (%)	1 (20)	41 (58.6)
Weight (kg)	10.1 ± 1.9	7.4 ±0.3
MUAC (cm)	13.3 ± 0.6	11.3 ± 0.2
Length (cm)	78.9 ± 6.5	75.9 ± 1.1
Weight- for-height, Z score	-0.2 ± 0.8	-3.1 ± 1.8
Kwashiorkor, n (%)	0	43 (61.4)
HIV positive, n (%)	0	31 (44.3)
Diarrhea within first 24 hr, n (%)	-	9 (19.6)
Hyponatremia,^1^ n (%)	0	10 (12.8)
Hypernatremia,^2^ n (%)	0	7 (9.0)
Duration of stay (days)	-	12 ± 4.8
Alanine aminotransaminase (IU/I) ϯ	14.0 (8.5–16.5)	45.5 (26.5–63.8)

Values are presented as mean ±SD, n (%) or median with interquartile range (IQR) as indicated by ϯ.

^1^ Hyponatremia defined as a plasma sodium concentration < 130 mmol/L

^2^ Hypernatremia defined as a plasma sodium concentration > 145 mmol/L

**Fig 1:**
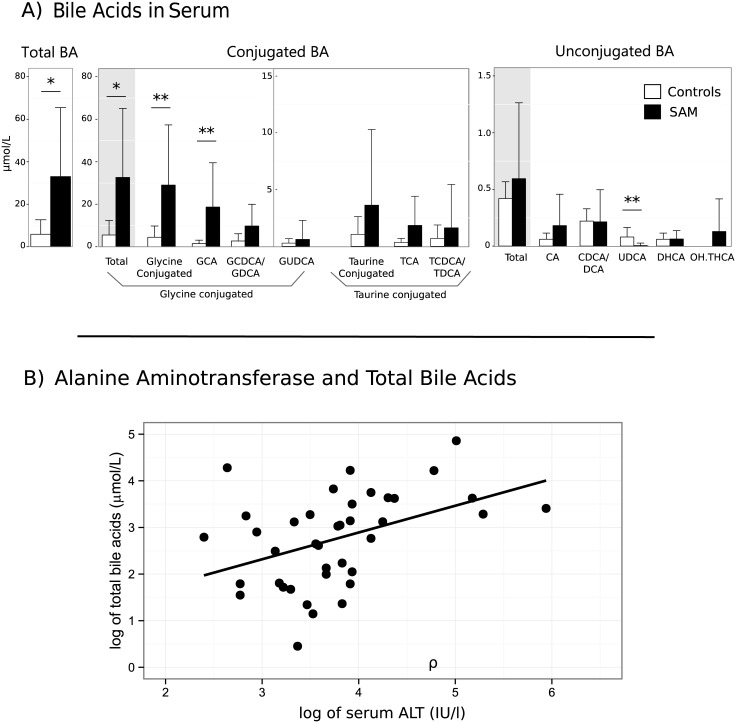
A) Bile acids accumulate in serum of SAM patients compared to controls in discovery cohort. Bar graphs represent mean and standard deviation (SD). Peaks for conjugated and unconjugated CDCA and DCA were indistinguishable and therefore presented as combined values (CDCA/DCA). Significant differences between healthy controls and patients with severe acute malnutrition (SAM) are indicated with stars; * *p*-value<0.05, ** *p*-value < 0.01 (Mann-Whitney test). B) Correlation between serum alanine aminotransaminase (ALT) and total bile acids concentrations in serum from the children with severe acute malnutrition of the follow up cohort. Values presented on log-scale. (Spearman correlation = 0.52, p<0.001).

Our discovery cohort showed that bile acid homeostasis is altered in SAM-patients compared to controls as detailed in Fig 1A. Serum bile acid profiles of malnourished children showed higher levels of total bile acids and this accumulation was mainly driven by conjugated bile acids. The glycine-conjugates were markedly elevated with a median (IQR) of 24.6 μmol/l [8.6–47.7] compared to 1.9 μmol/l [1.7–3.3] in controls (*p* = 0.01). Eleven children with SAM had detectable levels of C27 bile acids, *i*.*e*., trihydroxycholestanoic acid (THCA) and dihydroxycholestanoic acid (DHCA), but the total concentration was not significantly different from controls (S1 Table). Finally, there was a significant correlation between ALT and the concentrations of serum bile acids (ρ = 0.52, p<0.001) (Fig 1B). Even though 40% of our study cohort is HIV sero-positive, we did not find any significant effect of HIV on bile acids.

With the follow up cohort, we could analyze both serum and fecal samples on admission and prior to discharge in SAM-patients (Tables 2 and 3 and S2 and S3 Tables). Total serum concentration of bile acids did not differ between admission and pre-discharge with medians (IQR) of 18.4 μmol/l [5.1–33.2] and 16.6 μmol/l [8.0–33.8], respectively (Table 2). Yet, the ratio of conjugated to unconjugated bile acids increased significantly between admission and prior to discharge with median (IQR) of 43.9 μmol/l [21.8–120] compared to 116.1 μmol/l [55.3–457] (*p* <0.01). In fecal samples, conjugated bile acids were markedly lower 21.5 pmol/mg feces [6.7–119] to 148 pmol/mg feces [20.2–893] on admission compared to clinical recovery, i.e., prior to discharge. However, secondary unconjugated bile acids were significantly higher on admission going from 49.3 pmol/mg feces [3.2–1215] to 1.8 pmol/mg feces [0.6–8.9] (*p*<0.001) (Table 3). There were no consistent differences between bile acid profiles in children with kwashiorkor compared to marasmus.

**Table 2:** Serum bile acid concentrations in severely malnourished children at admission and prior to discharge in the follow up cohort.

	Admission	Pre-discharge	
Serum bile acids (mmol/L)	(n = 40)	(n = 40)	*p*-value
**Conjugated**	18.2 (5.0–32.2)	16.7 (8.1–33.3)	n.s
Glycine-conjugated	16.0 (4.7–26.1)	14.8 (7.1–31.9)	n.s
Taurine-conjugated	1.2 (0.5–3.2)	1.3 (0.6–2.7)	n.s
G/T ratio	9.4 (7.0–20.7)	12.2 (7.4–17.1)	n.s
**Unconjugated**	0.3 (0.1–0.6)	0.17 (0.05–0.35)	n.s
Primary bile acid (CA+CDCA)	0.3 (0.1–0.5)	0.1 (0.05–0.3)	n.s
Secondary bile acid (LCA+DCA)	0.01 (0.0–0.1)	0 (0.0–0.0)	n.s
Secondary/Primary Ratio	0.05 (0.0–0.3)	0 (0.0–0.0)	**<0.01**
**Conjugated/Unconjugated ratio**	43.9 (21.8–120)	116.1 (55.3–457)	**<0.01**
**Total bile acid**	18.4 (6.0–33.2)	17.0 (9.1–33.7)	n.s

Values expressed as median and interquartile range. Significant differences between admission and prior to discharge in patients with severe acute malnutrition (SAM) are indicated in bold, p<0.05 (Wilcoxon-signed Rank test). *p*-value >0.1 indicated as n.s. (not significant).

**Table 3:** Fecal bile acids concentrations during admission and at Pre-discharge.

	Admission	Pre-discharge	
Fecal bile acids (pmol/mg)	(n = 36)	(n = 37)	*p*-value
**Conjugated BA**	21.5 (6.7–119)	148 (20.2–893)	**<0.01**
**Unconjugated BA**	2761 (1792–5790)	2328 (1156–4565)	n.s
Primary bile acid (CA+CDCA)	1894 (563–5536)	2306 (978–4347)	n.s
Secondary bile acid (LCA+DCA)	49.3 (3.2–1215)	1.8 (0.6–8.9)	**<0.001**
Secondary/Primary Ratios	0.04 (0.0–1.2)	0.0 (0.0–0.01)	n.s
LCA/CDCA ratio	0.01 (0.0–1.2)	0.0 (0.0–0.001)	n.s
DCA/CA ratio	0.07 (0.0–1.2)	0.001 (0.0–0.01)	n.s
LCA	13.8 (0.9–387)	0.2 (0.01–1.2)	**<0.001**
DCA	23.1 (1.9–717)	1.4 (0.6–7.0)	**<0.01**
**Conjugated/Unconjugated ratio**	0.01 (0.0–0.08)	0.05 (0.01–0.4)	n.s
**Total bile acid**	2849 (1949–6214)	3425 (1978–5487)	n.s

Values expressed as median and interquartile range. Significant differences between admission and Pre-discharge in patients with severe acute malnutrition (SAM) are indicated in bold, p<0.05 (Wilcoxon-signed Rank test). *p*-value > 0.1 indicated as n.s. (not significant)

**Fig 2:**
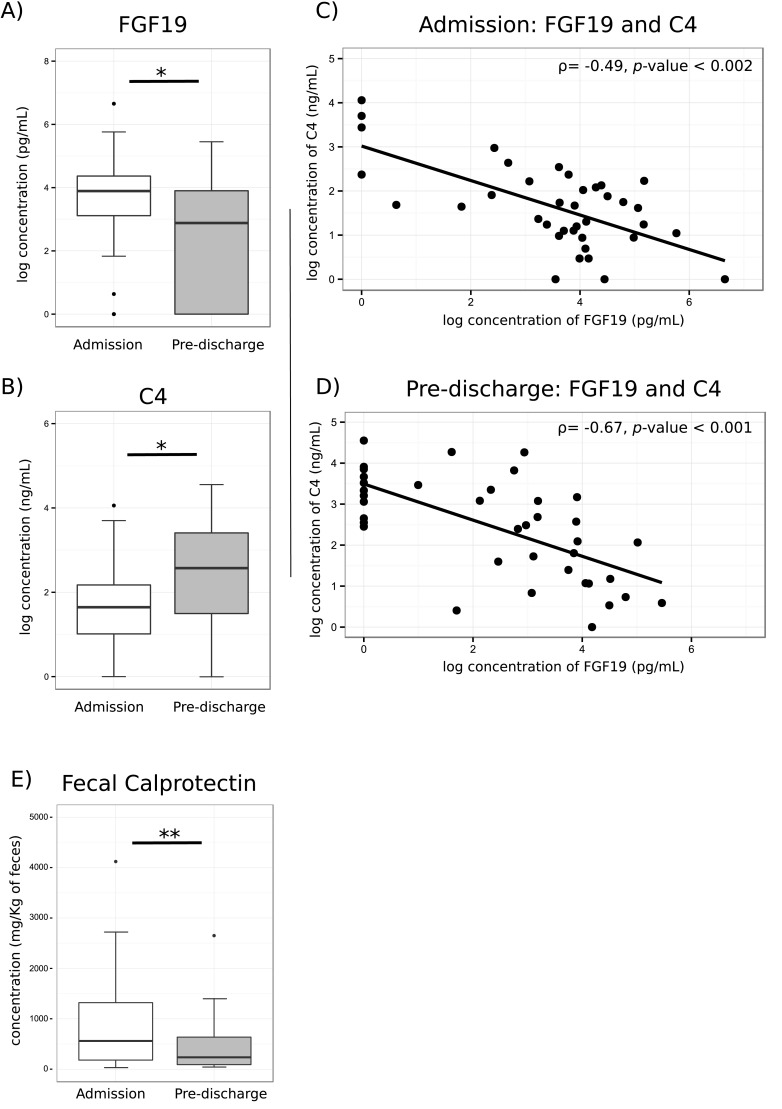
Differences in serum levels of A) FGF19 and B) C4 in children from the follow up cohort at admission and pre-discharge. FGF19 negatively correlates with serum concentration of C4 at C) admission and D) prior to discharge. E) Fecal calprotectin is decreased in children to be discharged. Boxplots represent 25th and 75th quartiles, box-midline indicates median and whiskers define top and bottom values within 1.5 times the inter-quartile range (IQR). Wilcoxon Signed Rank test was used to compare admission and pre-discharge for FGF19 and C4. Mixed effects models were used to test differences in calprotectin to account for missing values. Spearman test was used for correlation. Stars indicate; * *p*-value < 0.05, ** *p*-value < 0.01.

Markers of intestinal bile acid signaling (FGF19), and hepatic bile acid synthesis (C4) were altered between admission and pre-discharge. Serum FGF19 concentrations decreased from 48.0 [21.6–77.8] pg/ml to 16.9 [0–48.5] pg/ml (*p* = 0.007) after stabilization indicating higher intestinal bile acid signaling at admission (Fig 2A) and this was associated with lower C4 concentrations on admission compared to discharge, i.e., 4.2 ng/ml [1.8–7.8] and 12.1 ng/ml [3.5–29.3] (*p*<0.001) (Fig 2B). Levels of FGF19 negatively correlated with those of C4 both at admission (ρ = -0.49, *p*-value <0.002) and prior to discharge (ρ = -0.67, *p*-value <0.001) (Fig 2C and 2D). Fecal calprotectin, a marker of intestinal inflammation, was lower in children that were to be discharged (Fig 2E). However, there was no correlation between fecal bile acids, and the presence of diarrhea, or the fecal calprotectin levels.

## Discussion

Children with complicated SAM suffer from high inpatient and post-discharge mortality rates [27]. SAM has been associated with abnormal hepatic function [28], enteropathy [29] and intestinal dysbiosis [8]. As the liver, intestine and intestinal microbiota play a central role in regulating bile acid homeostasis, we hypothesized that these processes would be altered in children with SAM. Indeed, we showed that SAM is associated with increased serum bile acid levels, which remained elevated following standard nutritional intervention suggesting that normal bile acid homeostasis may not be attained in children deemed ready for discharge. They however show an increase in conjugated fecal bile acids, a decrease in serum FGF19 and increase in C4 levels.

The higher levels of total serum bile acids seen in children with SAM compared to controls could, in theory, reflect reduced hepatic bile acid uptake and hepatic biliary secretion, or increased re-absorption of bile acids at the level of the intestine. Two studies analyzing duodenal contents of children with SAM showed that SAM is associated with lower biliary concentration of particularly conjugated bile acids [20, 21]. Hepatic basolateral uptake of bile acids is mediated by the sodium/taurocholate co-transporting polypeptide (NTCP) and, to a lesser degree, solute carrier organic anion transporters (OATP’s). Expression of NTCP and OATP’s have been found to be reduced during hepatic inflammation or cholestasis as a protective mechanism [30] and might form an explanation for reduced duodenal bile acid concentrations in children with SAM. In addition, SAM is associated with bile acid malabsorption [31]. Our findings, along with these previously published studies suggest that increased serum bile acid levels most likely relate to reduced hepatic bile acid uptake and biliary secretion.

The lower levels of fecal conjugated bile acids that we found in children with SAM compared to pre-discharge was also demonstrated in a recent pre-clinical model of moderate malnutrition [4]. In conjunction with high levels of secondary bile acids in malnourished children on admission, these data suggest that potentially, dysbiosis associated with SAM [8] leads to increased deconjugation of bile acids. The latter renders them less bioavailable and, hence, more likely to pass into the colon where they can be converted from primary to secondary bile acids, through the action of certain bacteria. This has previously been attributed to bacterial overgrowth into the small intestine [32]. Finally, all children are treated with broad spectrum antibiotics which alters the microbiome and likely reduces the bacterial content in the small intestine. This could also lead to selective pressure against Clostridium leptum which can modify bile acids through 7α-dehydroxylation and de-conjugation inducing primary to secondary conversion [33, 34].

Bile acid synthesis via the classical pathway was reduced in children with SAM as indicated by lower C4 levels compared to pre-discharge. The nuclear bile acid receptor, FXR, highly expressed in the liver and intestine, regulates bile acid synthesis. The increased level of FGF19 during admission compared to pre-discharge could be related to an increased ileal presence of hydrophobic bile acids, which are stronger ligands for FXR [35]. Circulating FGF19 binds to FGFR4 in hepatocytes to negatively regulate bile acid synthesis in the liver by inhibiting the rate-limiting enzyme CYP7A1 [14]. Lower C4 levels in children with SAM were associated with higher FGF19 indicating increased inhibition of bile acid synthesis, which is an appropriate response considering the increased serum bile acid levels.

SAM has been associated with liver steatosis and inflammation [28]. In addition, serum ALT has been found to be especially increased in kwashiorkor thus indicating hepatic injury [36]. Elevated ALT is also found in hepatic steatosis or NAFLD-like phenotype [37]. It is known that hydrophobic bile acids can exert cytotoxic effects through the production of reactive oxygen/nitrogen species [38]. Their hepatotoxic impact causes stellate cells to proliferate and initiate apoptotic pathways [39, 40]. Bile acid induced hepatotoxicity has been implicated in the pathogenesis of liver injury in cholestatic liver diseases [41] and non-alcoholic fatty liver disease (NAFLD) [42]. Since we have found both a correlation between ALT and total bile acids and that malnutrition shifts the bile acid profiles towards a predominance of secondary bile acids, it would be important to determine whether these homeostatic changes play a role in the development of liver disease in SAM.

Similarly to the liver, bile acids can also cause intestinal injury. DCA and CDCA can promote apoptosis of colonocytes, with CDCA being more toxic [43]. Also, DCA and CDCA may enhance mucosal permeability and bacterial infiltration in enterocytes, which can further promote intestinal inflammation [17, 18]. DCA and CDCA have also been shown to increase intestinal permeability through epidermal growth factor receptor autophosphorylation, occludin dephosphorylation and re-arrangement of tight junctions [18]. We found that children close to being discharged had lower fecal calprotectin indicating that intestinal inflammation may be at least in part resolved. Our study, however, did not find a correlation between calprotectin, a marker of intestinal inflammation, and bile acids. This would need to be investigated further with histological data.

This study has several limitations. First, recruiting age-matched controls proved to be highly challenging in Malawi as caregivers were reluctant to provide multiple blood samples. It is very difficult to obtain consent from caregivers to take blood or stool from their children, especially if healthy due in part to cultural beliefs. We first aimed to determine whether bile acid concentrations are elevated in ill malnourished children. SAM changes liver metabolic function thus it is expected to also impact bile acid homeostasis. Unfortunately, we could not measure bilirubin levels since only small aliquots were available and stored samples had been exposed to variable amounts of light which would have affected measurements. We also did not have stool samples from healthy children to study the differences in bile acid elimination between healthy and malnourished children. In addition, the role of broad spectrum antibiotics was not determined as all children received protocolized antibiotics as per WHO treatment guidelines [44]. Mortality rate is up to 30% and infections are common, which explains the wide use of antibiotics in all children for at least 5 days. As bacteria play an important role in intestinal bile acid homeostasis, antibiotic treatment likely impact the results. These limitations and the fact that data was collected from two separate cohorts limits the ability to draw strong overarching conclusions. Despite these limitations, our results are still clinically informative given the extreme scarcity of data on bile acid homeostasis in severe acute malnutrition. However, we look forward to validating these results in future studies.

In conclusion, we provide evidence that bile acid homeostasis in children with SAM is significantly disturbed. These findings could have potential clinical implications relating to nutritional rehabilitation and interventions aimed at restoring intestinal and hepatic function in this highly vulnerable patient population.

## Supporting Information

S1 Table Serum bile acid concentration in severely malnourished children compared to healthy controls. (DOCX)

S2 Table Complete serum bile acid concentrations in severely malnourished children at admission and after clinical recovery. (DOCX)

S3 Table Complete fecal bile acid content in severely malnourished children at admission and after clinical recovery. (DOCX)
