# Supplementary material for: Impaired Bile Acid Homeostasis in Children with Severe Acute Malnutrition
Source: PLoS One. 2016 May 10;11(5):e0155143. doi: 10.1371/journal.pone.0155143 (PMC4862637; doi:10.1371/journal.pone.0155143)
Supplement: S1 Table — (DOCX) [file pone.0155143.s001.docx]

S1 Table. Serum bile acid concentration in severely malnourished children compared to healthy controls.

|  |  | **Controls** | | **SAM patients** | |  |
| --- | --- | --- | --- | --- | --- | --- |
| Bile acids (μmol/l) | | (n=5) | | (n=22) | | *p*-value |
| **Conjugated** | | 2.1 | (2.1-4.1) | 27.2 | (9.6-49.4) | **0.02** |
|  | Glycine-conjugated | 1.9 | (1.7-3.3) | 24.6 | (8.6-47.7) | **0.01** |
|  | GCA | 0.6 | (0.4-1.9) | 14.8 | (5.2-25.3) | **0.01** |
|  | GCDCA | 1.3 | (1.1-1.4) | 6.5 | (3.3-13.3) | 0.08 |
|  | GUDCA | 0.1 | (0-0.4) | 0.1 | (0-0.5) | n.s |
|  | Taurine-conjugated | 0.4 | (0.2-0.8) | 1.1 | (0.6-4.1) | n.s |
|  | TCA | 0.1 | (0.1-0.5) | 0.8 | (0.3-2.7) | 0.09 |
|  | TCDCA | 0.3 | (0.1-0.3) | 0.4 | (0.3-1.2) | n.s |
|  | G/T ratio | 4.3 | (4.3-9.5) | 12.7 | (6.2-27.3) | 0.07 |
|  |  |  |  |  |  |  |
| **Unconjugated** | | 0.4 | (0.4-0.5) | 0.4 | (0.2-0.9) | n.s. |
|  | CA | 0.1 | (0-0.1) | 0.1 | (0.1-0.2) | n.s. |
|  | CDCA | 0.2 | (0.2-0.2) | 0.1 | (0.1-0.2) | n.s |
|  | UDCA | 0.1 | (0-0.1) | 0 | (0-0) | **0.01** |
|  | DHCA | 0.1 | (0-0.1) | 0.05 | (0-0.1) | n.s |
|  | OH.THCA | 0 | (0-0) | 0 | (0-0.1) | n.s |
|  |  |  |  |  |  |  |
|  | C27 | 0.1 | (0-0.1) | 0.2 | (0.03-0.2) | n.s |
|  | C24 | 2.6 | (2.5-4.3) | 27.5 | (9.7-50.0) | **0.02** |
|  | C27/C24 | 0.04 | (0-0.04) | 0.004 | (0-0.02) | n.s |
|  |  |  |  |  |  |  |
| **Total BA** |  | 2.6 | (2.5-4.3) | 27.5 | (9.7-50.0) | **0.02** |

C29-dicarboxylic acid, TUDCA, tOH-THCA, THCA, tTHCA, tDHCA were below the detection

limit. Conjugated and unconjugated CDCA values include DCA signal as their peaks were

undistinguishable. Values expressed as median and interquartile range (IQR). BA, bile acids.

Significant differences between healthy controls and patients with severe acute malnutrition (SAM) are indicated in bold, p<0.05 (Mann-Whitney test). *p*-value > 0.1 indicated as n.s. (not significant).
