# Supplementary material for: Impaired Bile Acid Homeostasis in Children with Severe Acute Malnutrition
Source: PLoS One. 2016 May 10;11(5):e0155143. doi: 10.1371/journal.pone.0155143 (PMC4862637; doi:10.1371/journal.pone.0155143)
Supplement: S2 Table — (DOCX) [file pone.0155143.s002.docx]

S2 Table: Complete serum bile acid concentrations in severely malnourished children at admission and after clinical recovery

|  | **Admission** | | **Pre-discharge** | |  |
| --- | --- | --- | --- | --- | --- |
| **Serum bile acids (µmol/L)** | (n=40) | | (n=40) | | *p-value* |
| GCA | 8.3 | (1.9-15.9) | 6 | (12.3-13.5) | n.s. |
| TCA | 0.7 | (0.2-1.8) | 0.9 | (0.2-1.6) | n.s. |
| CA | 0.1 | (0.0-0.2) | 0.1 | (0.0-0.1) | n.s. |
| GCDCA | 3.8 | (1.2-8.1) | 3.7 | (2.1-7.4) | n.s. |
| TCDCA | 0.4 | (0.2-1.1) | 0.3 | (0.2-1.3) | n.s. |
| CDCA | 0.1 | (0.0-0.3) | 0.1 | (0.0-0.1) | 0.05 |
| GDCA | 0.2 | (0.0-0.5) | 0 | (0-0.1) | **0.001** |
| TDCA | 0 | (0.0-0.0) | 0 | (0.0-0.1) | 0.07 |
| DCA | 0 | (0.0-0.1) | 0 | (0.0-0.0) | **0.01** |
| GUDCA | 0.1 | (0.0-0.2) | 0 | (0.0-0.1) | **0.04** |

TUDCA, UDCA, GLCA, TLCA, LCA, GLCAS, TLCAS, TMCA, HDCA were below detection level. Values are expressed as median and interquartile range. Significant differences between admission and recovery in patients with severe malnutrition (SM) are indicated in bold, p<0.05 (Wilcoxon-signed Rank test). *p*-value > 0.1 indicated as n.s. (not significant).
