# Supplementary material for: Impaired Bile Acid Homeostasis in Children with Severe Acute Malnutrition
Source: PLoS One. 2016 May 10;11(5):e0155143. doi: 10.1371/journal.pone.0155143 (PMC4862637; doi:10.1371/journal.pone.0155143)
Supplement: S3 Table — (DOCX) [file pone.0155143.s003.docx]

S3 Table: Complete fecal bile acid content in severely malnourished children at admission and after clinical recovery

|  | **Admission** | | **Pre-discharge** | |  |
| --- | --- | --- | --- | --- | --- |
| **Fecal Bile acids (pmol/mg)** | (n=40) | | (n=40) | | *p*-value |
| TUDCA | 0.03 | (0.0-0.4) | 0.01 | (0.0-0.19) | n.s |
| GUDCA | 0.06 | (0.0-0.3) | 0.09 | (0.0-0.27) | n.s |
| TCA | 2.03 | (0.3-6.6) | 19.5 | (1.2-88.2) | **<0.01** |
| GCA | 4.2 | (0.9-37.5) | 28.8 | (6.8-335) | **<0.01** |
| TLCAS | 0.0 | (0.0-0.03) | 0.0 | (0.0-0.2) | 0.09 |
| GLCAS | 0.09 | (0.0-0.48) | 0.12 | (0.0-0.39) | n.s |
| CA | 1105 | (22-2605) | 1430 | (400-2270) | n.s |
| DCA | 23 | (1.9-718) | 1.4 | (0.6-7.0) | **<0.01** |
| LCA | 13.1 | (0.87-387) | 0.18 | (0.01-1.2) | **<0.001** |
| TCDCA | 0.75 | (0.08-13.4) | 14.3 | (0.3-79.3) | **0.01** |
| GCDCA | 3.8 | (1.6-33.3) | 19.6 | (6.0-233) | **<0.01** |
| GDCA | 0.2 | (0.04-1.9) | 0.3 | (0.05-2.1) | n.s |
| UDCA | 22.3 | (2.0-99.7) | 1.4 | (0.4-57.5) | n.s |
| HDCA | 0.8 | (0.08-8.1) | 0.1 | (0.0-0.3) | **<0.001** |
| CDCA | 771 | (105-2568) | 790 | (291-1507) | n.s |
|  |  |  |  |  |  |

TMCA, TLCA, GLCA, TDCA were below detection level. Values are expressed as median and interquartile range. Significant differences between admission and pre-discharge in patients with severe malnutrition (SM) are indicated in bold, p<0.05 (Wilcoxon-signed Rank test). *p*-value > 0.1 indicated as n.s. (not significant)
